# Data compilation regarding the effects of grain size and temperature on the strength of the single-phase FCC CrFeNi medium-entropy alloy

**DOI:** 10.1016/j.dib.2020.106712

**Published:** 2021-01-05

**Authors:** Mike Schneider, Guillaume Laplanche

**Affiliations:** Institute for Materials, Ruhr-University Bochum, Universitätsstr. 150, 44801 Bochum, Germany

**Keywords:** FeCrNi, Medium- and high-entropy alloys, Compression-test data, Tensile-test data, Density and average thickness of annealing twins, Hall-Petch parameters

## Abstract

In the present article, we present a data compilation reflecting recrystallized microstructures and the corresponding mechanical properties of an equiatomic, single-phase face-centered cubic (FCC) CrFeNi medium-entropy alloy (MEA). For the analysis, interpretation, and discussion of the data, the reader can refer to the original research article entitled “Effects of temperature on mechanical properties and deformation mechanisms of the equiatomic CrFeNi medium-entropy alloy”, see Ref. (*Schneider and Laplanche*, Acta Mater. 204, 2020). The data related to recrystallized microstructures comprise raw backscatter electron (BSE) micrographs (tif-files) obtained using a scanning electron microscope (SEM) for six grain sizes in the range [10–160 µm], optical micrographs of the alloy with the largest grain size (*d* = 327 µm), pdf-reports and tables presenting the corresponding grain-size distributions (*d*, accounting for grain boundaries only) and crystallite-size distributions (*c*, which accounts for both grain and annealing twin boundaries), the annealing twin thicknesses (*t*), the average number of annealing twin boundaries per grain (*n*), and the average Taylor factor (*M*) of each recrystallized microstructure. These are benchmark datasets that may serve to develop new algorithms for the automated evaluation of microstructural parameters. Such algorithms would help to speed up the analyses of microstructures and improve their reliability. Furthermore, several groups pointed out that in addition to the mean grain size, other microstructural parameters such as the grain size distribution (*Raeisinia* et al., Model. Simul. Mater. Sc. 16, 2008) and the average number of twins per grain (*Schneider* et al., Int. J. Plasticity, 124, 2020) may affect some material properties (e.g. Hall-Petch strengthening). Therefore, an effort was made here to determine and report almost all the microstructural parameters describing recrystallized microstructures of FCC alloys. The mechanical-properties data are provided as excel-sheets in which the raw stress-strain curves can be found. Compression tests for alloys with different grain sizes were performed at room temperature. Additional compression tests and tensile tests for the grain size *d* = 160 µm were performed at temperatures between 77 K and 873 K. Characteristic mechanical properties, such as yield stresses at 0.2% plastic strain (*σ_0.2%_*) and Hall-Petch parameters (*σ_0_* and *k_y_*) are given for all temperatures in the tables below. Moreover, the Hall-Petch parameters as well as the mechanical data reported in the present study could be used for data mining and implemented in programs used for alloy design.

## Specifications Table

**Table:** 

Subject	Materials Science
Specific subject area	High- and medium-entropy alloys (HEAs and MEAs), Austenitic stainless steels, Fe-based superalloys
Type of data	Micrographs (scanning electron microscopy and optical microscopy), Tables (microstructural parameters and Hall-Petch parameters), Excel-sheets (raw stress-strain curve data), pdf-files (assessment of grain- and crystallite sizes using the Heyn lineal intercept method)
How data were acquired	SEM: Quanta FEI 650 ESEM; OM: Zeiss Axio, Tensile/Compression testing machine: Zwick Roell XForce Z100
Data format	Raw (stress-strain curves, micrographs), Analyzed (grain/crystallite sizes, average annealing twin thicknesses, Taylor factors, Hall-Petch parameters)
Parameters for data collection	Backscatter electron images were obtained using an SEM of type Quanta FEI 650 ESEM with acceleration voltages between 15 kV and 30 kV and a working distance of 10 mm. Compression and tensile tests were performed at different temperatures with a constant strain rate of 10^–3^ s^–1^. Assessments of grain and crystallite sizes were carried out using the Heyn lineal intercept method.
Description of data collection	Metallographic samples were cut, embedded, and prepared by grinding, polishing, and etching.
Data source location	Institute for Materials, Ruhr-University Bochum, Universitätsstr. 150, 44,801 Bochum, Germany
Data accessibility	Data are available via https://data.mendeley.com/datasets/7d826s3mhf/1
Related research article	Schneider, M., Laplanche, G., 2021, Effects of temperature on mechanical properties and deformation mechanisms of the equiatomic CrFeNi medium-entropy alloy, Acta Materialia 204, 116470.

## Value of the Data

• Quantitative datasets of the recrystallized microstructures of an equiatomic, single-phase FCC CrFeNi medium-entropy alloy as well as its mechanical properties are reported here. These data may be useful for other researchers in the community of high- and medium-entropy alloys.
• The equiatomic CrFeNi alloy may also be interesting for researchers in the fields of austenitic stainless steels and Fe-based superalloys. Our data may improve the understanding of these complex engineering alloys and also help to further optimize them.
• The microstructural data compilation consists of BSE and optical micrographs of recrystallized FCC microstructures, tables and pdf-files reporting the corresponding grain/crystallite-size distributions, the thickness distribution of annealing twins and their density as well as the texture of the alloys. These data could be used to further improve the automated analysis of microstructures, e.g. algorithms for image analysis.
• Our mechanical raw-data (*i.e., stress strain curves*) could be used to further improve the automated analysis (*machine learning*) of yield stress, work hardening rate, ultimate tensile stress, homogeneous elongation and strain to fracture.
• The normalized Hall-Petch parameters (*relationship between yield stresses and grain/crystallite sizes*) reported here could be used to shed light on how these parameters are affected by chemistry, microstructure (*especially grain size distribution*), and alloy parameters such as the stacking fault energy and the shear modulus.

## 1 Data Description

**Fig. 1:**
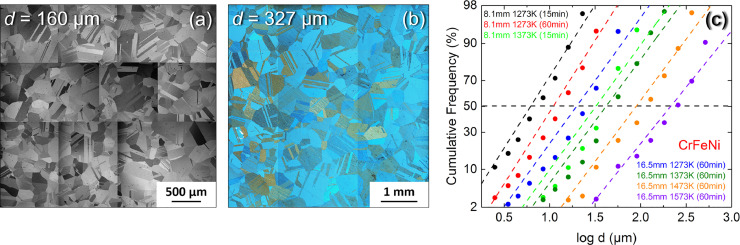
(a) Montage of nine BSE micrographs for the CrFeNi alloy with a mean grain size of 160 µm and (b) assembly of three optical micrographs used for the grain size assessment of the coarsest microstructure, *d* = 327 µm, and (c) logarithmic cumulative probability plots representative of the grain size distributions of all the alloys investigated in the present study after recrystallization anneals at temperatures between 1273 K and 1573 K and times ranging from 15 min to 60 min.

Since 2004, high- and medium-entropy alloys (HEAs and MEAs) have attracted tremendous attention in various scientific fields [1], [13], [2], [3], [4], [5], [6], [7], [8], [9], [10], [11], [12], [14]. However, the corresponding research data are not systematically reported in the literature, precluding data mining for further alloy development. The data compilation presented in the present article includes microstructural and mechanical data for the recrystallized, single-phase FCC, Cr_33.3_Fe_33.3_Ni_33.3_ (composition in at.%) medium-entropy alloy. Recrystallization heat treatments at temperatures lying in the range (1273 K–1573 K) for times between 15 min and 60 min yielded seven different recrystallized microstructures. BSE and optical micrographs of these microstructures were used in combination with the lineal intercept method to determine the grain- and crystallite-size distributions, see Fig. 1, Table 1, Table 2, Table 3, and pdf-reports in the linked Mendeley Data repository. Note that each pdf-report was obtained using the software (Imagic IMS Client V20H1) and contain a BSE image with overlaid test lines and intercepts. As this software is in german and that it is not possible to change the language to export the report, the most important data for the grain- and crystallite size distributions were translated and can be found in Tables 2 and 3, respectively, of the present article. In the present study, the mean grain/crystallite size is taken as the average intercept length. Following the standard test method ASTM E-112 [15], four equidistant and parallel test lines of identical length were used per micrograph. Four BSE micrographs spaced 1 mm apart were collected for each microstructure, except for the two coarsest. To meet the requirements of the standard test method ASTM E-112 [15] in these two latter cases, nine single BSE micrographs were assembled for the second coarsest microstructure while the alloy with the coarsest microstructure was etched to image its microstructure using optical microscopy. Fig. 1a shows the assembled BSE-micrograph while Fig. 1b displays a montage of three optical micrograph after etching.   The BSE micrographs can be either downloaded from https://data.mendeley.com/datasets/7d826s3mhf/1 or be sent on request by email. Fig. 1c shows a probability plot of the cumulative frequency vs. logarithm of grain diameter class for the seven heat treatments yielding different recrystallized microstructures. Note that a numerical linearization of the Gaussian distribution function was used on the scale of the *y*-axis in Fig. 1c. Besides the measurement of the average grain (*d*) and crystallite (*c*) sizes, the BSE and optical micrographs were also used to assess the number of annealing twin boundaries per grain (*n*) and the distribution of annealing twin thicknesses (*t*), which are reported in Table 1, Table 2, Table 3, Table 4, Table 5, respectively, with their respective uncertainties.

**Table 1:** Mean grain size (*d*), crystallite size (*c*) and average thickness of annealing twins (*t*) after heat treatments at different temperatures (*T*) and times of the CrFeNi alloy in the form of rods with two different diameters. Also listed are the magnifications used to image the recrystallized microstructures. The parameter *d* counts only the intersections between grain boundaries and the test lines, whereas *c* is determined by counting intersections with both grain and annealing twin boundaries.

rod diameter (mm)	*T* (K)	time (min)	*d* (µm)	*c* (µm)	*t* (µm)	Magnification
8.1	1273	15	10 ± 1	7 ± 1	2.9 ± 0.3	200
8.1	1273	60	19 ± 2	12 ± 1	4.9 ± 0.3	150
16.5	1273	60	34 ± 1	19 ± 1	8.6 ± 0.5	60
8.1	1373	15	55 ± 2	24 ± 1	12.3 ± 1.5	80
16.5	1373	60	75 ± 4	38 ± 1	18 ± 2	50
16.5	1473	60	160 ± 8	82 ± 4	31 ± 3	75
16.5	1573	60	327 ± 20	144 ± 10	52 ± 6	50

**Table 2:** Grain size distributions after heat treatments at different temperatures and times, see Table 1. These data were obtained from BSE and optical micrographs in combination with the linear intercept method. The grain sizes only accounts for the intersections of the test lines with grain-boundaries (annealing twin boundaries are excluded from the analysis). The mean grain size (*d*) with uncertainty (*∆d*) are shown at the bottom of the table in bold.

Size range	Absolute frequency						
0–2 µm	85	17	11	4	1	–	–
2–3 µm	112	26	6	1	1	–	–
3–4 µm	113	30	15	1	–	–	–
4–5 µm	146	36	15	5	2	–	–
5–7 µm	274	96	34	9	12	1	–
7–10 µm	312	151	59	17	13	1	–
10–13 µm	269	191	66	23	17	1	–
13–19 µm	309	286	151	63	30	2	1
19–27 µm	155	238	251	103	54	1	1
27–38 µm	44	209	279	154	114	13	2
38–75 µm	10	83	403	522	340	24	11
75–107 µm	–	3	81	185	232	22	6
107–151 µm	–	–	7	62	126	33	12
151–214 µm	–	–	–	7	41	31	17
214–302 µm	–	–	–	–	22	26	19
302–427 µm	–	–	–	–	5	16	25
427–600 µm	–	–	–	–	–	3	28
600 µm +	–	–	–	–	–	2	13

***d* (µm)**	**10**	**19**	**34**	**55**	**75**	**160**	**327**
***∆d* (µm)**	**1**	**2**	**1**	**2**	**4**	**8**	**20**

rod diameter (mm)	8.1	8.1	16.5	8.1	16.5	16.5	16.5
*T* (K)	1273	1273	1273	1373	1373	1473	1573
time (min)	15	60	60	15	60	60	60

**Table 3:** Crystallite size distributions after heat treatments at different temperatures and times, see Table 1. These data were obtained from BSE and optical micrographs in combination with the linear intercept method. The parameter (*c*) is determined by counting intersections with both grain and annealing twin boundaries. The mean grain size (*c*) with uncertainty (*∆c*) are shown at the bottom of the table in bold.

Size range	Absolute frequency						
0–2 µm	512	119	143	30	7	–	–
2– 3 µm	318	153	116	96	11	–	1
3–4 µm	266	135	129	125	54	–	–
4–5 µm	240	138	82	144	88	2	–
5–7 µm	400	253	239	233	117	6	1
7–10 µm	375	308	265	242	136	14	–
10–13 µm	241	273	214	236	97	11	5
13–19 µm	255	343	328	272	258	27	18
19–27 µm	105	208	355	351	248	25	22
27–38 µm	26	121	300	298	246	51	20
38–75 µm	4	49	291	452	473	71	63
75–107 µm	–	1	32	78	174	40	38
107–151 µm	–	–	–	23	71	42	34
151–214 µm	–	–	–	2	18	27	38
214–302 µm	–	–	–	–	4	24	25
302–427 µm	–	–	–	–	1	5	23
427–600 µm	–	–	–	–	–	1	12
600 µm +	–	–	–	–	–	–	6

***c* (µm)**	**7**	**12**	**19**	**24**	**38**	**82**	**114**
***∆c* (µm)**	**1**	**1**	**1**	**1**	**1**	**4**	**10**

rod diameter (mm)	8.1	8.1	16.5	8.1	16.5	16.5	16.5
*T* (K)	1273	1273	1273	1373	1373	1473	1573
time (min)	15	60	60	15	60	60	60

**Table 4:** Average number of annealing twin boundaries per grain (*n*) for different recrystallized microstructures. Also given are the mean grain/crystallite sizes.

*d* (µm)	10 ± 1	19 ± 2	34 ± 1	55 ± 2	75 ± 4	160 ± 8	327 ± 20
*c* (µm)	7 ± 1	12 ± 1	19 ± 1	24 ± 1	38 ± 1	82 ± 4	114 ± 10

*n* (−)	0.23	0.27	0.41	0.62	0.49	0.48	0.63
*∆n* (−)	0.01	0.01	0.03	0.03	0.05	0.01	0.02

**Table 5:** Twin thickness distributions after heat treatments at different temperatures and times (see Table 1) obtained from BSE and optical micrographs. The average twin thicknesses (*t*) with uncertainty (*∆t*) are shown at the bottom of the table in bold.

Size range	Absolute frequency						
0–2 µm	121	45	14	2	–	–	–
2–3 µm	60	52	25	1	1	1	–
3–4 µm	37	39	39	36	3	–	–
4–5 µm	31	31	23	37	4	–	–
5–7 µm	15	49	43	28	20	2	1
7–10 µm	13	43	47	42	27	9	–
10–13 µm	3	17	29	40	18	1	1
13–19 µm	1	2	36	27	42	5	6
19–27 µm	–	4	20	27	15	10	6
27–38 µm	–	–	3	17	16	15	9
38–75 µm	–	–	–	6	14	13	19
75–107 µm	–	–	–	–	3	3	5
107–151 µm	–	–	–	–	–	–	4
151–214 µm	–	–	–	–	–	–	2
214–302 µm	–	–	–	–	–	–	–
302–427 µm	–	–	–	–	–	–	–
427–600 µm	–	–	–	–	–	–	–
600 µm +	–	–	–	–	–	–	

***t* (µm)**	**2.9**	**4.9**	**8.6**	**12.3**	**18**	**31**	**52**
***∆t* (µm)**	**0.3**	**0.3**	**0.5**	**2**	**2**	**3**	**5**

rod diameter (mm)	8.1	8.1	16.5	8.1	16.5	16.5	16.5
*T* (K)	1273	1273	1273	1373	1373	1473	1573
time (min)	15	60	60	15	60	60	60

Besides the Heyn lineal intercept method applied to BSE and optical micrographs, electron backscatter diffraction (EBSD) was used to determine the mean grain and crystallite size distributions, see Tables 6 and 7. In this case, the mean grain and crystallite sizes were calculated using *d* = (*A*_d_ × π/4)^1/2^ (equivalent to the mean intercept length) and *c* = (*A*_c_ × π/4)^1/2^, where *A*_d_ and *A*_c_ are the average cross sectional areas of the grains and crystallites, respectively. Table 8 compares the mean grain and crystallite sizes obtained using EBSD and the Heyn lineal intercept method (LIM) for all recrystallized microstructures and shows the Taylor factors (*M*) determined by EBSD.

**Table 6:** Grain size distributions obtained by EBSD after heat treatments at different temperatures and times, see Table 1. The mean grain size (*d*_EBSD_) with uncertainty (*∆d*_EBSD_) are given at the bottom of the table in bold.

Size range	Absolute frequency						
0–2 µm	9	–	–	–	–	–	–
2–3 µm	4	2	–	–	–	–	–
3–4 µm	4	3	1	–	–	–	–
4–5 µm	7	–	–	–	–	–	–
5–7 µm	12	6	4	–	–	–	–
7–10 µm	18	11	7	–	8	–	–
10–13 µm	22	9	15	–	3	–	–
13–19 µm	15	21	20	3	13	–	–
19–27 µm	12	7	35	6	18	–	–
27–38 µm	7	8	32	9	30	2	–
38–75 µm	–	3	48	17	71	8	6
75–107 µm	–	–	3	4	25	2	2
107–151 µm	–	–	–	–	27	7	7
151–214 µm	–	–	–	–	15	6	8
214–302 µm	–	–	–	–	–	9	13
302–427 µm	–	–	–	–	–	3	9
427–600 µm	–	–	–	–	–	2	2
600 µm +	–	–	–	–	–	–	2

***d_EBSD_* (µm)**	**12**	**17**	**30**	**43**	**69**	**175**	**250**
***∆d_EBSD_* (µm)**	**2**	**2**	**4**	**5**	**7**	**10**	**25**

rod diameter (mm)	8.1	8.1	16.5	8.1	16.5	16.5	16.5
*T* (K)	1273	1273	1273	1373	1373	1473	1573
time (min)	15	60	60	15	60	60	60

**Table 7:** Crystallite size distributions determined by EBSD after heat treatments at different temperatures and times, see Table 1. These data were obtained by EBSD. The mean crystallite size (*c*_EBSD_) with uncertainty (*∆c*_EBSD_) are provided at the bottom of the table in bold.

Size range	Absolute frequency						
0–2 µm	137	–	–	–	–	–	–
2–3 µm	77	41	–	–	–	–	–
3–4 µm	99	30	29	–	–	–	–
4–5 µm	97	29	61	–	–	–	–
5–7 µm	80	59	77	12	–	–	–
7–10 µm	72	62	97	26	73	–	–
10–13 µm	30	58	98	24	81	–	–
13–19 µm	25	28	93	41	95	10	–
19–27 µm	5	12	105	30	98	16	–
27–38 µm	–	5	51	33	133	27	12
38–75 µm	–	2	29	21	212	67	63
75–107 µm	–	–	–	–	41	41	62
107–151 µm	–	–	–	–	11	22	35
151–214 µm	–	–	–	–	5	12	38
214–302 µm	–	–	–	–	–	3	11
302–427 µm	–	–	–	–	–	4	10
427–600 µm	–	–	–	–	–	–	3
600 µm +	–	–	–	–	–	–	–

***c_EBSD_* (µm)**	**5**	**8**	**15**	**21**	**35**	**79**	**116**
***∆c_EBSD_* (µm)**	**1**	**2**	**2**	**3**	**4**	**5**	**15**

rod diameter (mm)	8.1	8.1	16.5	8.1	16.5	16.5	16.5
*T* (K)	1273	1273	1273	1373	1373	1473	1573
time (min)	15	60	60	15	60	60	60

**Table 8:** Comparison of the mean grain (excluding twin boundaries) and crystallite (including twin boundaries) sizes obtained using the linear intercept method (*d_LIM_, c_LIM_*) with that determined by EBSD (*d_EBSD_, c_EBSD_*). Additionally given are the corresponding Taylor factors (*M*).

rod diameter (mm)	*T* (K)	time (min)	*d_LIM_* (µm)	*d_EBSD_* (µm)	*c_LIM_* (µm)	*c_EBSD_* (µm)	*M*
8.1	1273	15	10 ± 1	12 ± 2	7 ± 1	5 ± 1	3.08
8.1	1273	60	19 ± 2	17 ± 2	12 ± 1	8 ± 2	3.16
16.5	1273	60	34 ± 1	30 ± 4	19 ± 1	15 ± 2	3.03
8.1	1373	15	55 ± 2	43 ± 5	24 ± 1	21 ± 3	3.19
16.5	1373	60	75 ± 4	69 ± 7	38 ± 1	35 ± 4	3.03
16.5	1473	60	160 ± 8	175 ± 10	82 ± 4	79 ± 5	3.18
16.5	1573	60	327 ± 20	250 ± 30*	144 ± 10	116 ± 15*	3.10

⁎ The EBSD map for the alloy with a mean grain size of 327 µm contained only 60 grains while the size of 130 grains could be measured using the lineal intercept method on Fig. 1b.

To investigate the effect of grain refinement on mechanical properties, compression tests were conducted at 293 K for the seven grain sizes investigated in the present study. These data allowed us to plot the yield stress at 293 K as a function of the square root of the average grain/crystallite size. From these Hall-Petch plots, the intrinsic lattice strength (*σ*_0_) and the Hall-Petch slope (*k*_y_) were determined at room temperature following the procedure reported in Ref. [2], see Table 9. These values were then respectively normalized by *G* and *Gb*^1/2^, where *G* is the shear modulus and *b* is the Burgers vector. Both parameters were taken from Ref. [8]. The normalized Hall-Petch parameters (*σ*_0_/*G* and *k*_y_/(*Gb*^1/2^)) are reported in Table 10 as they allow to compare the strength and the magnitude of grain boundary strengthening of different alloys with the same crystallographic structure [16]. The temperature dependence of the yield stress was determined at seven additional temperatures (77 K, 173 K, 223 K, 373 K, 473 K, 673 K, and 873 K) for the CrFeNi alloy with a mean grain size of 160 µm, see Table 11.

**Table 9:** Hall-Petch parameters (*σ_0_* and *k_y_*) obtained at room temperature for the grain/crystalitte size datasets.

grain size dataset		crystallite size dataset	
*σ_0_* (MPa)	k_y_ (MPa µm^1/2^)	σ_0_ (MPa)	k_y_ (MPa µm^1/2^)
80 ± 8	966 ± 25	50 ± 3	897 ± 40

**Table 10:** Normalized Hall-Petch parameters (*σ_0_*/*G* and *k_y_/Gb^1/2^*) at room temperature for the grain/crystallite size datasets. The shear modulus, *G*, and Burgers vector, *b*, were taken from Ref. [8].

grain size dataset		crystallite size dataset			
(σ_0_/G) × 1000 (MPa)	k_y_/Gb^1/2^ (-)	(σ_0_/G) × 1000 (MPa)	k_y_/Gb^1/2^ (-)	G (GPa)[8]	b (nm)[8]
1.01 ± 0.05	0.76 ± 0.04	0.63 ± 0.04	0.71 ± 0.04	79.3	1.466

**Table 11:** Compression yield stresses σ_0.2%_ for seven grain (*d*) and crystallite (*c*) sizes obtained at eight different temperatures.

		σ_0.2%_ (MPa)							
*d* (µm)	*c* (µm)	77 K	173K	223K	293 K	373K	473K	673 K	873 K
10 ± 1	7 ± 1	–	–	–	359 ± 2	–	–	–	–
19 ± 2	12 ± 1	–	–	–	286 ± 9	–	–	–	–
34 ± 1	19 ± 1	–	–	–	261 ± 9	–	–	–	–
55 ± 2	24 ± 1	–	–	–	213 ± 2	–	–	–	–
75 ± 4	38 ± 1	–	–	–	185 ± 3	–	–	–	–
160 ± 8	82 ± 4	359 ± 12	231 ± 35	162 ± 12	149 ± 7	154 ± 7	125 ± 4	91 ± 2	83 ± 25
327 ± 20	144 ± 10	–	–	–	163 ± 8	–	–	–	–

The Excel-sheets containing the corresponding stress-strain data can be found in the linked Mendeley Data repository under the “CrFeNi_Compression_Tests”-folder. This folder is divided into eight subfolders corresponding to the eight testing temperatures. The Excel-sheets in these folders are named using the three following characteristics: alloy composition, recrystallization heat treatment (temperature and time), and temperature of the compression test. For instance, the Excel-sheet for a compression test conducted at 473 K with a CrFeNi alloy that was recrystallized at 1473 K for 60 min is labelled as: “CrFeNi_1473 K_60min_473 K”. From the stress-strain datasets, the yield stresses at 0.2% plastic deformation (*σ*_0.2%_) determined at different temperatures for various grain and crystallite sizes are given in Table 11.

Additional tensile tests were performed at six different temperatures (77 K, 173 K, 223 K, 293 K, 373 K, and 473 K) on samples with a given grain size of *d* = 160 µm. The resulting raw stress-strain data can be found in the linked Mendeley Data repository under the “CrFeNi_Tensile_Tests”-folder. The structure of the subfolders and the naming of the Excel-sheets is the same as for the compression tests. From these stress-strain datasets, the yield stresses at 0.2% plastic strain (*σ*_0.2%_), the ultimate tensile stresses (UTS), the uniform elongation (*ε*_uniform_), and the elongation to fracture (*ε*_fracture_) determined at different temperatures for a given grain/crystallite size are reported in Table 12. Please note, that in the case of the *ε*_uniform_ and *ε*_fracture_ values, those marked with an asterisk were determined from the crosshead displacement and subsequently corrected by a correction factor (∼0.8) while the other values were directly estimated using an extensometer. For the detailed description of this procedure, the reader may refer to the related research article [1].

**Table 12:** Temperature dependence of the tensile yield stress, σ_0.2%_, ultimate tensile stress, UTS, uniform elongation, *ε*_uniform_, and elongation to fracture, *ε*_fracture_, for the recrystallized CrFeNi alloy with a mean grain/crystallite size of *d* = 160 µm and *c* = 82 µm, respectively.

*T* (K)	*σ*_0.2%_ (MPa)	UTS (MPa)	*ε*_uniform_ (%)	*ε*_fracture_ (%)
77	370 ± 10	875 ± 12	44 ± 4	45± 3
173	254 ± 7	630 ± 26	42 ± 1*	46 ± 1*
223	198 ± 10	571 ± 4	40 ± 1*	44 ± 1*
293	174 ± 9	512 ± 3	36 ± 1	40 ± 1
373	175 ± 2	482 ± 20	29 ± 1*	35 ± 2*
473	126 ± 12	431 ± 7	36 ± 1*	40 ± 1*

## 2 Experimental Design, Materials and Methods

Except for the alloy with the coarsest grain size (*d* = 327 µm), the recrystallized materials were ground with SiC abrasive papers, polished with diamond suspensions, and vibropolished in a mixture of distilled water and colloidal silica (particle size: 0.06 µm). BSE micrographs were recorded in an SEM of type Quanta FEI 650 ESEM using a working distance of ∼10 mm. Acceleration voltages between 15 kV (small grains) and 20 kV (large grains) were chosen to optimize the BSE contrast. Four BSE images spaced 1 mm apart were collected for each grain size, except for the two coarsest microstructures. Here, two different methods were used to obtain micrographs covering sufficiently large surface areas. In the first method used for the second-largest grain size (*d* = 160 µm), nine BSE micrographs were collected and assembled, covering an area representative of the whole cross-section of a compression specimen, see Fig. 1a. In the second method employed for the largest grain size (*d* = 327 µm), the sample was etched using a Kalling II etching solution prior to imaging. This solution consists of 100 ml ethanol, 100 ml hydrochloric acid (32 vol.% in distilled water) and 5 g copper chloride. The specimen was hold in this solution for ∼5 s. The etched specimen was then rinsed, dried and observed in an optical microscope of type Zeiss Axio and three optical micrographs were mounted together and are shown in Fig. 1b.

The BSE and optical micrographs were then used to determine the mean grain (*d*) and mean crystallite (*c*) sizes along with their corresponding distributions using the Heyn lineal intercept method with four horizontal and four vertical lines, see Tables 2 and 3. Each line intersected ∼50 grains resulting in 300-500 intercepts per micrograph, similar to the procedure reported in Ref. [2]. The same procedure was used to determine the size distribution of the thicknesses of annealing twins, which is reported in Table 5 including the mean values (*t*) and corresponding uncertainties (∆*t*). Using the data for *d* and *c* and the equation *n* = (*d*/*c* - 1), the average number of annealing twin boundaries per grain (*n*) was calculated, see Table 4, similar to the procedure reported in Refs. [17,18].

Grain orientation maps were determined by electron backscatter diffraction (EBSD) in the above-mentioned SEM equipped with a Hikari XP camera (EDAX, AMETEK). From these orientation maps, grain and crystallite size distributions (*d*_EBSD_, *c*_EBSD_, see Tables 6 and 7, respectively) and Taylor factors (*M*, see Table 8) were determined . Evaluation of the data was performed using the TSL OIM Analysis (version 6.2.0) software (see Refs. [17,18]). Table 8 compares the results of the two previously mentioned methods, namely the Heyn lineal intercept method performed on BSE and optical micrographs (*d*_LIM_, *c*_LIM_) and the EBSD method (*d*_EBSD_, *c*_EBSD_). Please note that in the latter case grain and crystallite sizes were calculated using d = (A_d_×π/4)^1/2^ and c = (A_c_×π/4)^1/2^, which assumes that grain and crystallites have an equiaxed geometry.

Compression and tensile tests were conducted in a Zwick Roell XForce Z100 machine at temperatures ranging from 77 K to 873 K and at a nominal strain rate of 10^−3^ *s* ^−1^ for both deformation modes. To minimize friction between the compression samples and punches, the faces of both were lubricated with a MoS_2_ grease. The compression specimens were deformed up to true plastic strains ranging between 16% and 22%, while tensile tests were either conducted until rupture or interrupted at various plastic strains ranging between 5% and 20%. Tensile tests at 77 K and 293 K were performed with the aid of an axial extensometer (Model 3442, *Epsilon Technology Corp.*) directly attached to the gage section. For the other temperatures, a different method was used since the extensometer could not be used in these cases. Here, the strains were directly calculated from the crosshead displacement and corrected by a correction factor. The correction factor was determined by analyzing the tensile data obtained at 77 K and 293 K using either the strains determined with the extensometer and those determined from the crosshead displacement. At 77 K and 293 K, the strains calculated using the cross-head displacement were found to represent 80% of the strains determined with the extensometer, resulting in a correction factor of ∼0.8. The resulting values are marked with an asterisk in Table 12.

## CRediT Author Statement

**Mike Schneider:** Methodology, Investigation, Writing - Original Draft; **Guillaume Laplanche:** Writing - Review & Editing, Supervision.

## Declaration of Competing Interest

The authors declare that they have no known competing financial interests or personal relationships, which have, or could be perceived to have, influenced the work reported in this article.

## References

[1] Schneider M., Laplanche G. (2020). Temperature dependence of mechanical properties and deformation mechanisms in an equiatomic CrFeNi medium-entropy alloy. Acta Mater..

[2] Schneider M., George E.P., Manescau T.J., Záležák T., Hunfeld J., Dlouhý A., Eggeler G., Laplanche G. (2020). Analysis of strengthening due to grain boundaries and annealing twin boundaries in the CrCoNi medium-entropy alloy. Int. J. Plast..

[3] Wu Z., Bei H., Pharr G.M., George E.P. (2014). Temperature dependence of the mechanical properties of equiatomic solid solution alloys with face-centered cubic crystal structures. Acta Mater..

[4] Schneider M., Werner F., Langenkämper D., Reinhart C., Laplanche G. (2019). Effect of temperature and texture on hall–petch strengthening by grain and annealing twin boundaries in the MnFeNi medium-entropy alloy. Metals.

[5] Otto F., Dlouhý A., Somsen C., Bei H., Eggeler G., George E.P. (2013). The influences of temperature and microstructure on the tensile properties of a CoCrFeMnNi high-entropy alloy. Acta Mater..

[6] Laplanche G., Schneider M., Scholz F., Frenzel J., Eggeler G., Schreuer J. (2020). Processing of a singlecrystalline CrCoNi mediumentropy alloy and evolution of ist thermal expansion and elastic stiffness coefficients with temperature. Scr. Mater..

[7] Ding Q., Zhang Y., Chen X., Fu X., Chen D., Chen S., Gu L., Wei F., Bei H., Gao Y., Wen M., Li J., Zhang Z., Zhu T., Ritchie R.O., Yu Q. (2019). Tuning element distribution, structure and properties by composition in high-entropy alloys. Nature.

[8] Laplanche G., Gadaud P., Bärsch C., Demtröder K., Reinhart C., Schreuer J., George E.P. (2018). Elastic moduli and thermal expansion coefficients of medium-entropy subsystems of the CrMnFeCoNi high-entropy alloy. J. Alloys Compd..

[9] Couzinié J.-.P., Senkov O.N., Miracle D.B., Dirras G. (2018). Comprehensive data compilation on the mechanical properties of refractory high-entropy alloys. Data Brief.

[10] Gorsse S., Nguyen M.H., Senkov O.N., Miracle D.B. (2018). Database on the mechanical properties of high entropy alloys and complex concentrated alloys. Data Brief.

[11] Zhang B., Gao M.C., Zhang Y., Guo S.M. (2015). Supporting data for senary refractory high-entropy alloy CrxMoNbTaVW. Data Brief.

[12] Asabre A., Pfetzing-Micklich J., Stryzhyboroda O., Kostka A., Hecht U., Laplanche G. (2019). Data regarding the influence of Al, Ti, and C additions to as-cast Al0.6CoCrFeNi compositionally complex alloys on microstructures and mechanical properties. Data Brief.

[13] Raeisinia B., Sinclair C.W., Poole W.J., Tomé C.N. (2008). On the impact of grain size distribution on the plastic behaviour of polycrystalline metals. Model. Simul. Mater. *Sci.*.

[14] Tapia A.J.S.F., Yim D., Kim H.S., Lee B.-.J. (2018). Data to reproduce and modify “An approach for screening single phase high-entropy alloys using an in-house thermodynamic database”. Data Brief.

[15] American Society for Testing and Materials (ASTM) (2004). ASTM E112-10: Standard Test Methods for Determining Average Grain Size.

[16] Cordero Z.C., Knight B.E., Schuh C.A. (2016). Six decades of the Hall-Petch effect – a survey of grain-size strengthening studies on pure metals. Int. Mater. Rev..

[17] Schneider M., Werner F., Langenkämper D., Reinhart C., Laplanche G. (2020). Data compilation on the effect of grain size, temperature, and texture on the strength of a single-phase FCC MnFeNi medium-entropy alloy. Data Brief.

[18] Schneider M., George E.P., Manescau T.J., Záležák T., Hunfeld J., Dlouhý A., Eggeler G., Laplanche G. (2019). Benchmark dataset of the effect of grain size on strength in the single-phase FCC CrCoNi medium entropy alloy. Data Brief.

